# FY-3D MERSI On-Orbit Radiometric Calibration from the Lunar View

**DOI:** 10.3390/s20174690

**Published:** 2020-08-20

**Authors:** Ronghua Wu, Peng Zhang, Na Xu, Xiuqing Hu, Lin Chen, Lu Zhang, Zhongdong Yang

**Affiliations:** 1 Hefei Institutes of Physical Science, Chinese Academy of Sciences, Hefei 230031, China; wurh@cma.cn; 2 University of Chinese Academy of Sciences, Beijing 100049, China; 3 Key Laboratory of Radiometric Calibration and Validation for Environmental Satellites, National Satellite Meteorological Center, Beijing 100081, China; xuna@cma.cn (N.X.); huxq@cma.cn (X.H.); chenlin@cma.cn (L.C.); zhanglu11@stu.xjtu.edu.cn (L.Z.); yangzd@cma.cn (Z.Y.)

**Keywords:** lunar irradiance, Medium Resolution Spectral Imager (MERSI), reflective solar bands (RSBs), FY-3

## Abstract

Limited by the on-orbital calibration capability, scaling the measured radiance in accuracy and stability is challenging for the Earth observation satellites in the reflective solar bands (RSBs). Although the lunar calibration is a well-developed technique in the RSBs, limited work has been done for Chinese Earth observation satellites. To improve the on-orbital calibration performance, the advanced MEdium Resolution Spectral Imager (MERSI II), which is the primary payload of the fourth satellite of the Fengyun 3 Series (FY-3D), expands the space view angle of the imager in order to capture better lunar images. In this study, we propose an absolute radiometric calibration method based on the FY-3D/MERSI lunar observation data. A lunar irradiance model named ROLO/GIRO has been used together with the necessary data procedures, including dark current count estimation, single pixel irradiance calculation, and full disk lunar irradiance calculation. The calibration coefficients obtained by the lunar calibration are compared with the pre-launch laboratory calibration. The results show that the deviations between the two calibration procedures are in a reasonable range in general. However, a relatively high non-linear response was found in the low energy incidence for some detectors, which leads to the large deviation in the corresponding bands. This study explored an ideal and independent method to validate MERSI on-orbit radiometric performance. The lunar calibration practiced for MERSI also gave a valuable example that can be recommended to the other Chinese Earth observation satellites.

## 1. Introduction

Radiometric calibration is critical for the quantification of changes in the Earth observation from the satellite. However, scaling the measured radiance in accuracy and stability is still a challenging process. It is more practical in the wavelength of the reflective solar bands (RSBs) because of the limited capability of the on-orbital facility in this spectral region. From the satellite field of view, the Moon is stable in radiance [1] and has been treated as an ideal natural reference to calibrate the satellite measurements [2,3]. Few studies have been done for the lunar calibration of the Chinese Earth observation satellite since there has been no project to perform the active lunar view from the satellite platform since 2013 [4].

The MEdium Resolution Spectral Imager (MERSI) is one of primary payloads onboard the Chinese FengYun 3 (FY-3) meteorological series satellite [5,6]. The advanced MERSI (MERSI II) on the FY-3D is an upgraded version of the MERSI on the FY-3A/3B/3C series [7]. MERSI II has six additional infrared bands compared with the latter, which enhances its capability to detect surface and atmospheric elements in more detail. In addition, the MERSI II expands the space view angle of the imager in order to capture better lunar images to perform the lunar calibration.

Using the radiometric calibration coefficient, the MERSI on-orbit output digital number (DN) can be converted to a reflectance or radiance value. In the pre-launch phase, a traceable spherical integrating source is used as the radiometric reference to calibrate the MERSI RSBs in the visible (VIS) and near infrared (NIR) spectra in the laboratory [8]. In the on-orbit operational phase, multiple methods are used to calculate the calibration coefficients, which include Deep Convective Clouds (DCC) [9], Pseudo-Invariant Calibration Sites (PICS) [10], Simultaneously Nadia Observation (SNO) [11], etc., together with the pre-launch calculated results in the laboratory. The lunar observation data can be used as a radiometric reference in order to calibrate the MERSI RSBs. Compared with the existed methods, there are three major advantages of the lunar calibration, including stability of the lunar reflection, which has a fluctuation rate of about 10^−8^/year [12,13]; low-brightness target for solar reflection bands with a reflectivity of less than 10%; and singular observation target without stray light scattered by the Earth’s atmosphere.

The moon is an ideal target for the space-based instruments in the moderate resolution. The SeaWifs uses lunar data to monitor the radiometric stability over its lifetime, and the biases of lunar calibrations are 2–3% [14]. Both the scheduled and unscheduled lunar data of MODIS are used for monitoring the on-orbit changes of detector gains [15]. Choi [16] and Xiong [3] estimate the on-orbit performance of the Suomi National Polar-orbiting Partnership Visible Infrared Imaging Radiometer Suite (NPP/VIIRS) using the lunar data independently. High resolution remote sensors such as Hyperion [17] also perform the calibration procedure based on the lunar viewing data. Chinese LIBRA will take the moon as an important target to support inter-calibration with radiometric traceability [18].

The space view (SV) field angle for MERSI on the FY-3C satellite was increased to enable it to acquire full-disk moon images. This was the first time that the FY-3 series satellite imager could capture full-disk moon images. Wu [4] used the full-disk moon images data to track the attenuation of the radiometric calibration coefficient. The ratio between the irradiances of the target channel and the reference channel was proposed as the key parameter to monitor the radiometric performance of MERSI on the orbit. In particular, the ratios in time series are regressed to correct the attenuation of the MERSI’s radiometric response without the help of the lunar irradiance model. Nevertheless, this method relies on the stability of the reference channel. Otherwise, the attenuation of the target channel’s radiometric response will be affected by the fluctuations of the reference channel.

Furthermore, in order to obtain the absolute radiometric calibration coefficient, a lunar irradiance model should be used. For the VIS and NIR spectrum, the two existing models, i.e., the Miller and Turner Model (MT2009) [19] and the RObotic Lunar Observatory (ROLO) models, are both widely used. The MT2009 model is primarily used for quantitative product inversion for the day/night band, while the ROLO model, which was developed by the United States Geological Survey (USGS), is used for remote sensor calibration. Zhang [20] compared the two models using earth-based observations. The obtained results indicate that ROLO is more consistent with the earth-based observations rather than MT2009. In addition, Zhang [21] improved the accuracy of the ROLO model for NIR spectrum.

The Global Space-based Inter-Calibration System (GSICS), which is sponsored by the World Meteorological Organization, is used to inter-calibrate global remote sensing instruments and associate them with a common reference. They have developed the GSICS Implement of ROLO (GIRO) in order to define a common unique calibration reference for the international community. Considering it is recommended by GSICS, the GIRO model developed from the ROLO model was selected as the radiometric reference to implement the MERSI lunar calibration in this study. Based on the ROLO model, Zhang [22] discussed a cross-calibration method among multiple sensors, which could validate one satellite instrument with a reference instrument bridged by the moon.

FY-3D/MERSI was launched in November 2017. Its SV samples are twice as much as FY-3C/MERSI, which improves the qualities of lunar observation data [23]. Based on the on-orbit lunar observation data and the GIRO model, we propose an absolute radiometric calibration method for MERSI. Details of the MERSI lunar calibration method are introduced, especially for those personalized problems, such as fluctuating dark current DN and oversampling in the scanning direction of MERSI, which are different from MODIS lunar calibration. The first lunar observation data are used as the case study to illustrate the details of the lunar calibration method. Then the results from the lunar calibration are verified for the consistency compared with ground laboratory data. The nonlinear response phenomenon of band 19 for low energy incidence is then discovered through this method. Since the other methods, such as DCC, PICS, etc., can only specify the characteristics in the medium and high energy incidence, the lunar calibration provides a supplementary approach to understand the behaviors of the MERSI in the whole dynamic range of the radiometric response.

The remainder of this paper is organized as follows. Section 2 describes the lunar observation data and lunar irradiance model used in our study. The lunar calibration method used to obtain the MERSI absolute calibration coefficient is introduced in Section 3. Section 4 discusses the obtained results and compare them with the pre-launch measurement values. Finally, Section 5 presents our conclusions.

## 2. Lunar Observation Data and Lunar Irradiance Model

### 2.1. MERSI Lunar Observation Data

MERSI uses a 45° scanning mirror to obtain 25 bands data, and the spectral configuration and key specifications of FY-3D MERSI are shown in Table 1. Four types of the output digital number (DN) for each scan contains information from the Earth view (EV), blackbody (BB), on-board calibrator (OBC), and space view (SV) components. The BB, OBC, and SV data are used to meet the requirements of the on-board radiometric calibration system. Based on these data, the EV DN can be transformed to reflectance or radiance values for further quantitative applications.

**Figure 1:**
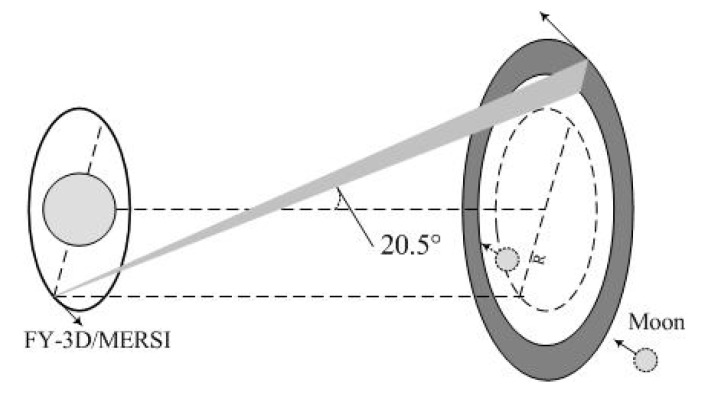
Schematic diagram of the geometric relationship between the moon and the FengYun 3D meteorological satellite (FY-3D) MEdium Resolution Spectral Imager (MERSI).

MERSI lunar observation is realized through the SV field, which is set at 69.5° from the nadir and 90° clockwise to the direction in which the satellite is moving. In general, the SV DN is used to provide dark current measures for calibration. However, at a special geometric angle, which is shown in Figure 1, the moon could travel through the SV field, during which lunar radiance information can be recorded. Thus, in the case of MERSI, lunar observations are unscheduled, unlike in the case of MODIS, which uses a scheduled maneuver to obtain observation data for calibration.

Figure 1 depicts the geometric arrangement for the MERSI lunar observation. As the satellite revolves around the Earth, the SV is scanned as a torus in space (concentric ring). Because the period of revolution for the moon is about 29 days, the moon passes through the scanned torus once about every month. Unfortunately, because of that, the relative geometrical relationship between the sun-synchronous satellite orbital plane and moon’s orbital plane cannot satisfy the geometrical restriction. For FY-3D MERSI, June, July, August, and September, the moon will be “upper” for the conic part of the space view depicted in Figure 1, so the moon cannot pass through the conic. It is different from MODIS, which can observe the moon by satellite maneuvering in this condition. Therefore, MODIS can obtain lunar data twelve times per year. MERSI does not carry out active maneuvers, so it can only obtain about eight moon observations per year for the remaining months.

MERSI is a multi-detector parallel scan remote sensor with two resolution settings. The 250-m resolution band is combined with 40 detectors, which means that 40 lines of scan data are obtained simultaneously for each frame scan at this resolution. In contrast, 10 lines of scan data are simultaneously obtained for the 1000-m resolution band. Overall, through the SV field, MERSI captures 192 and 48 samples per line for the 250-m and 1000-m resolution bands, respectively. Bands 1–4 are 250-m resolution bands, whereas the other bands are 1000-m resolution bands.

FY-3D MERSI got its first lunar observation data with good data quality on 29 December 2017. As a typical study case, these data are used to demonstrate the performance of the lunar calibration method to MERSI.

**Figure 2:**
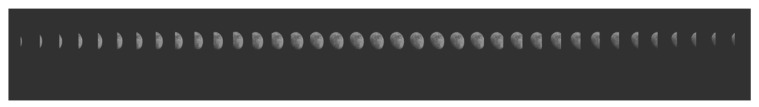
Moon images captured by FY-3D/MERSI (Band 1; 29 December 2017).

Due to the relative motion between the moon and satellite, a set of SV moon images are captured. In these images, the moon appears at different positions between scans as it moves from one side to the other of the scanned torus; this is shown in Figure 2. In particular, Figure 2 shows the images of the moon observed through band 1 on 29 December 2017.

For one single frame scan, the viewing angle of the 40-detector system is about 0.6888°; thus, the viewing angle of the 192 acquired samples is about 2.26°, whereas the solid angle of the moon is about 0.5° for an observer on Earth. This indicates that MERSI can obtain the moon images for all bands using a single frame via SV. Thus, it satisfies the calibration requirement wherein an image of the entire moon is required to calculate lunar irradiance. Based on the integrity of the moon disk in the images, the frame data of the SV images, whose bands contain the full-disk moon, are selected and extracted.

**Figure 3:**
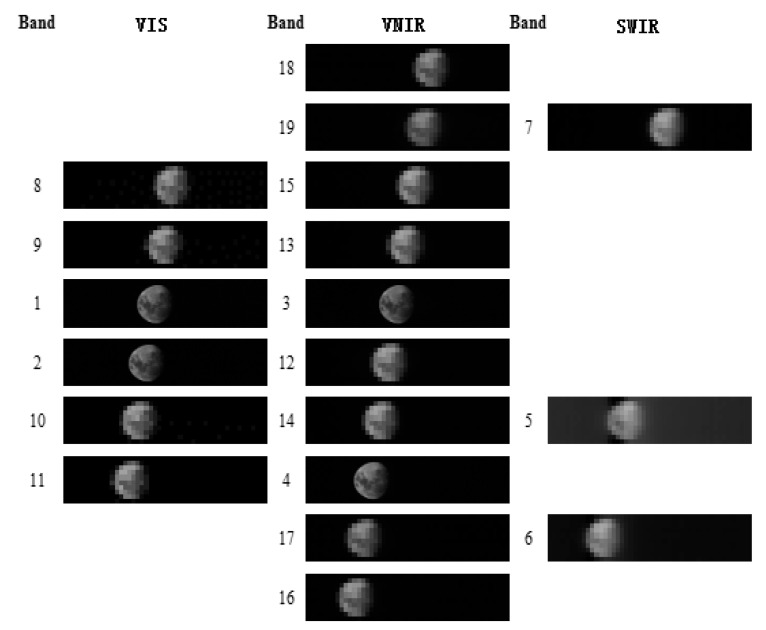
Lunar images by MERSI (29 December 2017).

**Figure 4:**
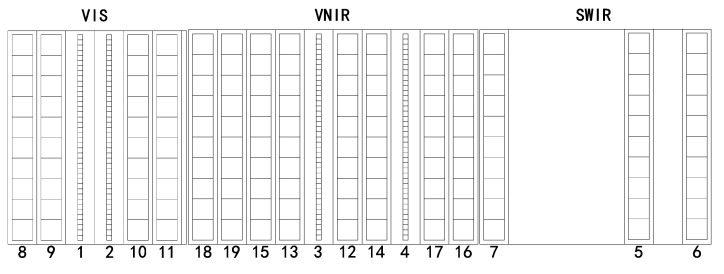
MERSI focal plane assemblies (FPAs). The visible (VIS) FPA comprises bands 8, 9, 1, 2, 10, and 11; the visible and near-infrared (VNIR) FPA comprises bands 18, 19, 15, 13, 3, 12, 14, 4, 17, and 16; and the short-wave infrared (SWIR) FPA comprises bands 7, 5, and 6.

Figure 3 shows the SV moon images of the MERSI solar reflectance bands. The images are aligned based on their positions on the Focal Plane Assemblies (FPAs). Although the samples of the SV image for the 250-m and 1000-m resolution bands are different, their observation angle is the same, and all bands images are converted to the same size as is shown in the figure. In particular, MERSI groups its RSBs on three FPAs, which contain the visible (VIS, 0.412–0.555 μm) bands, the visible and near-infrared (VNIR, 0.65–1.03 μm) bands, and the short-wave infrared (SWIR, 1.38–2.14 μm) bands in Figure 4. VIS FPA comprises bands 8, 9, 1, 2, 10, and 11; VNIR FPA comprises bands 18, 19, 15, 13, 3, 12, 14, 4, 17, and 16; and SWIR FPA comprises bands 7, 5, and 6. When the 45° scanning mirror is rotated, the incident optical line scans the detector array consecutively according to the detector array position, and all bands simultaneously record the DN. Therefore, the moon appears at different positions for the SV images of different bands.

### 2.2. Lunar Irradiance Model

As previously specified, in our study, we use GIRO [24] as the standard radiometric reference source, which is a common and validated implementation of the GSICS lunar radiometric reference. The GIRO model is a spectral response function (SRF) content driven model, which means that it involves cycles on the bands in the spectral range of 0.3–2.5 µm.

GIRO requires the observation time, the location of the sensor, the selenographic longitude of the Sun, and the selenographic latitude and longitude of the observer as inputs to calculate the absolute phase angle of the moon. Finally, the lunar spectral irradiance for the bands described by the SRFs of the sensor can be outputted.

With GIRO, satellite agencies can use the same lunar irradiance model to calibrate their remote sensing instruments. As a consequence, the accuracies of these instruments can be easily compared with each other.

**Figure 5:**
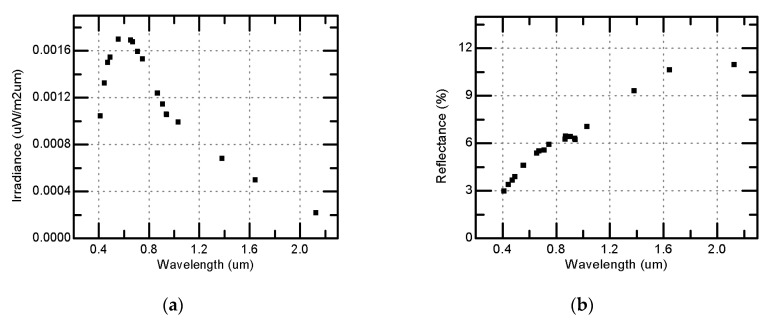
Lunar irradiance and reflectance for MERSI bands simulated by GIRO (29 December 2017). (**a**) *X*-axis is wavelength, and *Y*-axis is irradiance; (**b**) *X*-axis is wavelength, and *Y*-axis is reflectance.

For FY-3D/MERSI, the first lunar observation occurred on 29 December 2017, and the corresponding lunar observation geometry is listed in Table 2. The lunar irradiance and equivalent reflectance for MERSI 19 bands from the GIRO simulation are shown in Figure 5. The lunar phase angle is approximately 50 degrees when the MERSI collected lunar image data and the lunar equivalent reflectance are smaller 10% for the VNIR bands, especially for short wavelength bands. Therefore, the lunar data are suitable for describing the radiometric response in the low dynamic range of the incident energy.

## 3. MERSI Lunar Irradiance and Absolute Calibration

### 3.1. Single Pixel Irradiance

For MERSI, the irradiance of a single pixel can be calculated as follows:

(1) $$I_{pixel}=\omega L=\omega \left[k\left(DN-DC\right)Es/\pi \right]$$

where $I_{pixel}$ is the observation irradiance and ω is the instantaneous field of view (IFOV), which is (0.3 mrad)^2^ and (1.2 mrad)^2^ for the 250-m and 1000-m resolution bands, respectively. L is the single pixel observation radiance. k is the MERSI calibration coefficient, and DN is the observation digital count. In addition, DC is the dark current count, which is typically set to the SV DN value. Es is the band solar constant, which is the convolutional result between the SRF and spectral solar constant. Thuillier’s data are used as the reference solar constant for the MERSI laboratory measurements, for which ROLO uses Wehrli’s data. The spectrum difference between Thuillier’s data and Wehrli’s data is about 1.4% in the VIS and NIR spectrum [25]. This different will be included in the deviation between the results based on these two sources data.

### 3.2. Lunar Full-Disk Irradiance

The full-disk irradiance of the moon is calculated by integrating all the moon pixels over the SV image. Considering that the image is discrete data, the integral sign is replaced by the sum sign as follows:

(2) $$I_{moon}=f_{os}\sum_{moon}I_{pixel}$$

where $I_{moon}$ is the full-disk lunar irradiance, $I_{pixel}$ is the single pixel irradiance, and $f_{os}$ is the oversampling factor.

### 3.3. Lunar Calibration Coefficient

Combining Equations (1) and (2), the calibration coefficient k for MERSI can be derived as follows:

(3) $$k_{}=\frac{I_{moon}}{f_{os}\omega Es/\pi \sum_{moon}\left(DN-DC\right)}$$

where $I_{moon}$ is the lunar irradiance obtained from the GIRO model, and the other symbols have the same meaning as previously explained. The parameters $f_{os}$ and DC are important and will be explained in more details in the following subsections.

### 3.4. Oversampling Factor

The full-disk moon image can be captured using two methods. First, as is shown in Figure 3, the full-disk moon image can be captured using one frame and multiple detectors, and this image is referred to as a single-frame multi-detector image. Second, a full-disk moon image, referred to as multi-scan single-detector image, can be captured by extracting different frame data using a single detector, and then combining them to form a single image.

In both types of images, an oversampling effect has been considered in two orthogonal directions, namely the detector array direction and scan sample direction. The oversampling effect means that two adjacent pixels are overlapping for some same parts. The energy of the pixels is overestimated and needs to be corrected by the oversampling factor. The oversampling factor here is defined as the percent of the effective area after removing the overlapping area. In the case of oversampling in the scan sample direction, corresponding to the width of the moon image, a 27% overlap is observed between two sample intervals, which indicates that the oversampling factor in this direction ($f_{os-scan}$) is 0.73. In contrast, in the detector array direction and corresponding to the height of the moon image, the oversampling factor ($f_{os-detector}$) is equal to 1.0 for single-frame multi-detector images, whereas for other types of moon images, its value depends on the geometrical arrangement of the moon, satellite, and sun. For MERSI, the lunar irradiance from a single-frame multi-detector moon image is more suitable for radiance calibration than others. Therefore, the oversampling factor $f_{os}$ equals $f_{os-scan}$ × $f_{os-detector}$, which for the value used in Equation (3) is 0.73.

### 3.5. Dark Current Count

In order to estimate the DN response in the incident of lunar irradiance, it is necessary to eliminate the dark current count from the observed SV DN of MERSI. Considering that the moon is a low-brightness target, (DN-DC) for a MERSI lunar pixel is almost the same order of magnitude as the DC value. The accuracy of the dark current count has a significant influence on the calibration coefficient.

**Figure 6:**
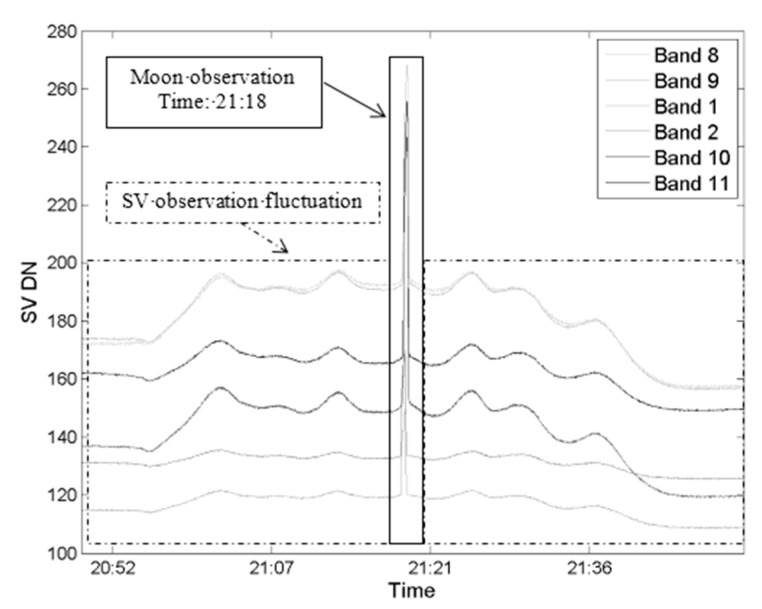
Space view (SV) data around the lunar observation (29 December 2017).

When the moon is being observed through the SV field, the dark current count cannot be estimated directly. The dark current calculated from the SV observation fluctuates on the basis of the position of the satellite in this paper. The SV observations for the RSBs of the MERSI on 29 December 2017, are shown in Figure 6. Considered the fluctuation in the SV observations, it is very important to calculate the DC from the fluctuating SV count for MERSI and other similar sensors. We use the data from 50 frames before and after the moon appeared in the SV field to calculate the SV DN average value as the dark current count value. Based on the lunar observation data from 29 December 2017, the DCs are listed in Table 3. These values are compared with those obtained from the pre-launch laboratory measurements.

The on-orbit working conditions of the detectors are different from those used in the ground laboratory test, such as the FPA temperature. At the same time, the SV field may be polluted by stray light, which increased the observed DC count to be higher than the pre-launch measurements. Both of these two effects will result in the large DC deviation. From Table 3, the DC deviations are larger than 20 counts at band 5, 6, 7, 15, and 19. The large DC deviation cannot be ignored because it may lead to the large bias in the calibration procedure. This effect will be discussed in Section 4.

## 4. Calibration Results and Discussion

In this section, the lunar calibration results for all of the FY-3D MERSI RSBs are compared with the pre-launch calibration, which was conducted in the laboratory based on the traceable spherical integrating sources (SIS). With these SISs, the full dynamic characterizations of the MERSI in the RSBs are described in the laboratory. However, the ambient environment has been changed after the launch. It leads to the on-orbit performance, which always differs from the laboratory. It is vital to validate the calibration status on the orbit with an independent method such as the lunar calibration.

### 4.1. Calibration Results

**Figure 7:**
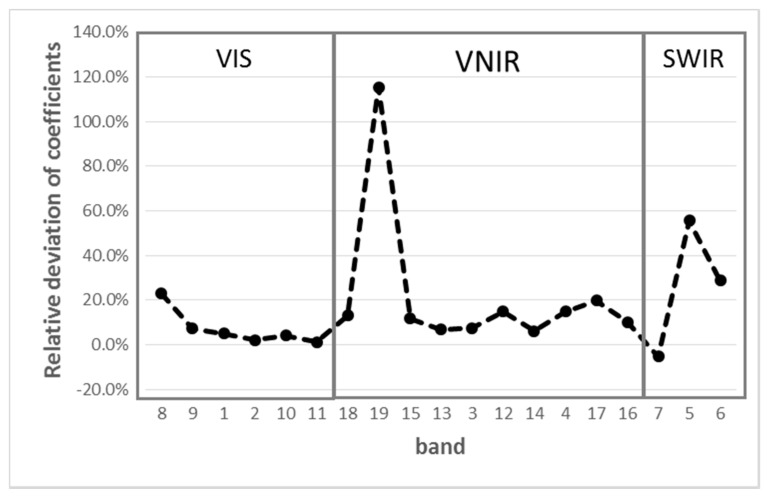
Relative differences of coefficients (classified based on FPA). *X*-axis is the MERSI band number, and *Y*-axis is the relative differences of coefficients, which ranges from −20% to 140%.

The lunar calibration was conducted using 29 December 2017 data, and the results are listed in Table 4. As a comparison, the pre-launch results as well as their relative deviation are also listed. The relative deviations range from 1.3% to 115.3%. The lunar calibration coefficient is diverse according to the different FPAs, as shown in Figure 7. For VIS FPA, the consistency between the lunar calibration and prelaunch measurement are better than 7.2%, and the mean value of the relative deviation is 3.2%, except for band 8. For VNIR FPA, the relative deviations vary from 6.3% to 115.3%, and the mean relative deviation is 10.6%, except for bands 17 and 19, whose relative deviations are relatively larger than others. For SWIR FPAs, the results of the lunar calibration are relatively poor, and the reason will be discussed later in this paper. As a summary, the consistency between the lunar and pre-launch calibration is within 10% for most of the bands except for band 19 and the bands in SWIR.

### 4.2. Discussion for the SWIR Bands

The detectors of the SWIR bands are made from the photovoltaic (PV) HgCdTe materials and work at 130 K ambient temperature on the orbit. The pre-launch calibration is conducted in a small vacuum tank to simulate the on-orbital conditions. However, the environment cannot be congruent completely, which will lead to the bias. We found that the DC deviations between the lunar calibration and the pre-launch calibration results in the SWIR bands are obviously lager than in the other bands. Especially for the band 7 (refer to the Table 3), the DC from the lunar observation is 144.1, almost 2.5 times the data from the laboratory, whose value is 59.9. This phenomenon is partly due to changes in the on-orbit conditions after launch, and partly due to the crosstalk contamination. As explored in [26], there are significant signal crosstalk phenomena for MERSI SWIR bands, and the crosstalk signal can reach to 40%. Therefore, the application of the lunar calibration method to MERSI SWIR is not recommended.

### 4.3. Discussion for the NIR Bands

**Figure 8:**
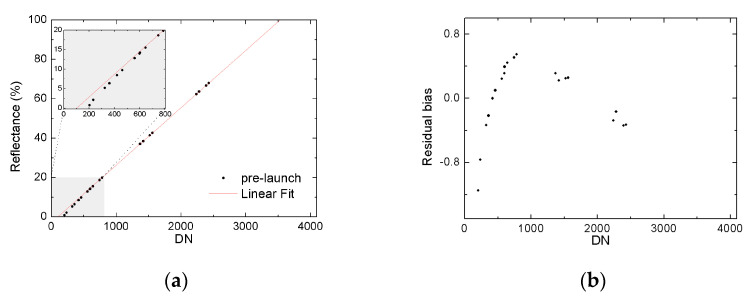
Pre-launch laboratory calibration measurements distribution of Band 19. (**a**) *X*-axis is the MERSI response digital number (DN), and *Y*-axis is the reference reflectance; (**b**) *X*-axis is the MERSI response DN, and *Y*-axis is the residual bias.

The lunar calibration is an efficient method to assess the radiometric performance of the instrument in the low dynamic range since the typical lunar reflectance is less than 0.1. In Figure 7, the lunar calibration result of band 19 (1.03 μm) is approximately two times larger than that from the laboratory. The deviation is far beyond the normal range. It was found that this large calibration deviation comes from the non-linear effect of detectors. The pre-launch results of the nonlinear radiometric response for the band 19 are shown in Figure 8, where *X*-axis is the MERSI response DN, and *Y*-axis is the reference reflectance in Figure 8a and the residual bias in Figure 8b.

The pre-launch calibration was fitted based on the whole dynamic measurements in Figure 8a. The calibration coefficient is the slope of the linear regression drawn in red line. The real measured data during the pre-launch phase depart from the regression line, especially when the DN is smaller than 400. The residual bias in Figure 8b shows the clear nonlinear distributed pattern with the variation of the DN number.

The center spectral of the band 19 is at the edge of spectral response of silicon detectors, which typically range from 0.4 μm to 1.0 μm. At the edge of the spectrum, the detector response signal is quite weak, which is more difficult for non-linear control. Similarly, band 8 (0.412 μm) also faces a similar problem. Generally, the lunar calibration is helpful to find the nonlinear effect by supplying the low reflectance information for the MERSI. In addition, if we desire a full dynamic calibration, the other vicarious calibration methods can be introduced based on integrated calibration [27], such as DCC, PICS, and RadCalNet [28].

## 5. Conclusions

Scaling the measured radiance in accuracy and stability in the RSBs of Fengyun satellites is challenging work since the performance of the OBC is poor. As a stable natural target, the moon has been used as a radiometric traceable reference to calibrate the satellite measurements. In this study, a lunar calibration approach has been proposed to be implemented for the FY-3D/MERSI. From the SV field of MERSI, the lunar observation data have been obtained. With the ROLO/GIRO model as the radiometric reference, the MERSI calibration coefficient is calculated.

The results from the lunar calibration are compared with the pre-launch laboratory measurements. Generally, the good consistency (within 10%) between the lunar and pre-launch calibration is obtained for the most RSB of the MERSI except the band 19 and bands in the SWIR. Considering the changed orbital conditions after the launch and the crosstalk contamination, the lunar calibration method is not recommended for the MERSI SWIR bands. As the center spectral of the band 19 is at the edge of the spectral response of the silicon detectors where the performance is hard to control, the relatively high non-linear radiometric response was found by the lunar calibration in this paper.

As a conclusion, this study explored an ideal and independent method to validate the MERSI on-orbit radiometric performance. The lunar calibrated results remain encouragingly consistent with those from the pre-launch laboratory test for the most MERSI RSBs. The lunar calibration implemented to the MERSI also gave a valuable example that can be recommended to the other Chinese Earth observation satellites. In the future, all the lunar observation data since the FY-3D MERSI working on-orbit will be processed using the method described in this paper, and the results would be compared with other methods results, such as DCC, PICS, or RadCalNet.

## Figures and Tables

**Table 1:** Bands and key specifications of the FengYun 3D meteorological satellite (FY-3D) MEdium Resolution Spectral Imager (MERSI).

Band	Center Wavelength (μm)	Band Width (nm)	Nadir FOV (m)	L_typ_^1^/T_typ_	SNR ^2^/NEdT	Dynamic Range
b01	0.470	50	250	35.3	100	0–90%
b02	0.550	50	250	29.0	100	0–90%
b03	0.650	50	250	22	100	0–90%
b04	0.865	50	250	25	100	0–90%
b05	1.38	30	1000	6	100	0–90%
b06	1.64	50	1000	7.3	200	0–90%
b07	2.13	50	1000	1.2	100	0–90%
b08	0.412	20	1000	44.9	300	0–30%
b09	0.490	20	1000	32.1	300	0–30%
b10	0.555	20	1000	36.8	500	0–30%
b11	0.670	20	1000	27.8	500	0–30%
b12	0.709	20	1000	19.2	500	0–30%
b13	0.746	20	1000	24	500	0–30%
b14	0.865	20	1000	17.8	500	0–30%
b15	0.905	20	1000	22.2	200	100%
b16	0.936	20	1000	20	100	100%
b17	0.940	50	1000	15.0	200	100%
b18	1.03	20	1000	5.4	100	100%
b20	3.8	180	1000	300	0.25	200–350 K
b21	4.050	155	1000	300	0.25	200–380 K
b22	7.2	500	1000	270	0.30	180–280 K
b23	8.550	300	1000	270	0.25	180–300 K
b24	10.8	1000	250	300	0.4	180–330 K
b25	12.0	1000	250	300	0.4	180–330 K

^1^ Unit for L_typ_ is W/(m^2^ μm sr); Unit for T, NEdT is K. ^2^ SNR and L_typ_ are specifications for reflective solar bands; NedT and T_typ_ are specifications for thermal emissive bands.

**Table 2:** Date of the observations and geometry.

Date	Absolute Phase Angle	Selenographic Longitude of the Sun	Selenographic Latitude of the Observer	Selenographic Longitude of the Observer
29 December 2017	44.3°	39.5°	−4.5°	6.7°

**Table 3:** Dark current (DC) values based on lunar calibration and prelaunch laboratory calibration.

Band	Pre-Launch DC	Lunar Calibration DC	DC Deviation
b01	128.0	124.8	−3.2
b02	134.6	131.0	−3.5
b03	248.4	247.8	−0.6
b04	143.6	148.6	5.0
b05	120.6	144.4	23.8
b06	79.5	126.0	46.5
b07	59.9	144.1	84.2
b08	195.5	195.5	0.0
b09	181.1	199.5	18.5
b10	136.1	155.7	19.6
b11	171.4	166.9	−4.5
b12	186.5	203.4	16.8
b13	198.4	204.0	5.6
b14	195.5	198.9	3.4
b15	150.5	172.6	22.1
b16	173.2	171.6	−1.6
b17	237.0	238.5	1.5
b18	237.2	239.0	1.8
b19	137.4	197.3	60.0

**Table 4:** Calibration coefficient based on lunar calibration and prelaunch laboratory calibration.

Band	Pre-Launch Coefficient	Lunar Calibration Coefficient	Relative Deviation
b01	0.02542	0.02671	5.1%
b02	0.02682	0.02742	2.2%
b03	0.02783	0.02992	7.5%
b04	0.02629	0.03010	14.5%
b05	0.02581	0.04016	55.6%
b06	0.02313	0.02978	28.8%
b07	0.02401	0.02275	−5.3%
b08	0.008452	0.010386	22.9%
b09	0.008869	0.009510	7.2%
b10	0.008430	0.008789	4.3%
b11	0.010830	0.010972	1.3%
b12	0.009302	0.010683	14.8%
b13	0.008765	0.009360	6.8%
b14	0.008661	0.009204	6.3%
b15	0.008634	0.009650	11.8%
b16	0.02717	0.02997	10.3%
b17	0.02745	0.03292	19.9%
b18	0.0286	0.03233	13.0%
b19	0.0295	0.06351	115.3%

## References

[1] Kieffer H.H., Stone T.C. (2005). The Spectral Irradiance of the Moon. Astron. J..

[2] Eplee R.E., Meister G., Patt F.S., Barnes R.A., Bailey S., Franz B.A., McClain C.R. (2012). On-orbit calibration of SeaWiFS. Appl. Opt..

[3] Xiong X., Sun J., Fulbright J., Wang Z., Butler J.J. (2015). Lunar Calibration and Performance for S-NPP VIIRS Reflective Solar Bands. IEEE Trans. Geosci. Remote Sens..

[4] Wu R.H., Zhang P., Yang Z.D., Hu X.Q., Ding L., Chen L. (2016). Monitor the radiance calibration of the remote sensing instrument by using the reflected lunar irradiance. J. Remote Sens..

[5] Zhang P., Yang J., Dong C., Lu N., Yang Z., Shi J. (2009). General introduction on payloads, ground segment and data application of Fengyun 3A. Front. Earth Sci. China.

[6] Yang Z., Zhang P., Gu S., Hu X., Tang S., Yang L., Xu N., Zhen Z., Wang L., Wu Q. (2019). Capability of Fengyun-3D Satellite in Earth System Observation. J. Meteorol. Res..

[7] Zhang P., Lu Q., Hu X., Gu S., Yang L., Min M., Chen L., Xu N., Sun L., Bai W. (2019). Latest Progress of the Chinese Meteorological Satellite Program and Core Data Processing Technologies. Adv. Atmos. Sci..

[8] Xu N., Niu X., Hu X., Wang X., Wu R., Chen S., Chen L., Sun L., Ding L., Yang Z. (2018). Prelaunch Calibration and Radiometric Performance of the Advanced MERSI II on FengYun-3D. IEEE Trans. Geosci. Remote Sens..

[9] Chen L., Hu X., Xu N., Zhang P. (2013). The Application of Deep Convective Clouds in the Calibration and Response Monitoring of the Reflective Solar Bands of FY-3A/MERSI (Medium Resolution Spectral Imager). Remote Sens..

[10] Wang L., Hu X., Chen L., He L. (2018). Consistent Calibration of VIRR Reflective Solar Channels Onboard FY-3A, FY-3B, and FY-3C Using a Multisite Calibration Method. Remote Sens..

[11] Xu N., Chen L., Wu R., Hu X., Sun L., Zhang P. (2014). In-flight intercalibration of FY-3C visible channels with AQUA MODIS. In Proceedings of the Earth Observing Missions and Sensors: Development, Implementation, and Characterization III. SPIE Int. Soc. Opt. Eng..

[12] Holsclaw G.M., McClintock W.E., Domingue D.L., Izenberg N.R., Blewett D.T., Sprague A.L. (2010). A comparison of the ultraviolet to near-infrared spectral properties of Mercury and the Moon as observed by MESSENGER. Icarus.

[13] Kieffer H.H. (1997). Photometric Stability of the Lunar Surface. Icarus.

[14] Eplee J.R.E., Barnes R.A., Patt F.S., Meister G., McClain C.R. (2004). SeaWiFS Lunar Calibration Methodology after Six Years on Orbit. Earth Observing Systems IX.

[15] Xiong X., Angal A., Twedt K.A., Chen H., Link D.O., Geng X., Aldoretta E., Mu Q. (2019). MODIS Reflective Solar Bands On-Orbit Calibration and Performance. IEEE Trans. Geosci. Remote Sens..

[16] Choi T., Shao X., Cao C. (2018). On-orbit radiometric calibration of Suomi NPP VIIRS reflective solar bands using the Moon and solar diffuser. Appl. Opt..

[17] McCorkel J., Thome K., Ong L. (2012). Vicarious Calibration of EO-1 Hyperion. IEEE J. Sel. Top. Appl. Earth Obs. Remote Sens..

[18] Zhang P., Lu N., Li C., Ding L., Zheng X., Zhang X., Hu X., Ye X., Ma L., Xu N. (2020). Development of the Chinese Space-Based Radiometric Benchmark Mission LIBRA. Remote Sens..

[19] Miller S., Turner R. (2009). A Dynamic Lunar Spectral Irradiance Data Set for NPOESS/VIIRS Day/Night Band Nighttime Environmental Applications. IEEE Trans. Geosci. Remote Sens..

[20] Zhang L., Zhang P., Hu X.Q., Chen L., Wang Y., Wang W. (2017). Comparison of lunar irradiance models and validation of lunar observation on Earth. J. Remote Sens..

[21] Zhang L., Zhang P., Hu X., Chen L., Min M. (2017). A novel hyperspectral lunar irradiance model based on ROLO and mean equigonal albedo. Optik.

[22] Zhang L., Zhang P., Hu X., Chen L., Min M., Xu N., Wu R. (2019). Radiometric Cross-Calibration for Multiple Sensors with the Moon as an Intermediate Reference. J. Meteorol. Res..

[23] Wu R.H., Zhang P., Zheng X.B., Hu X.Q., Xu N., Zhang L., Qiao Y.L. (2019). Data collection and irradiance conversion of lunar observation for MERSI. Opt. Precis. Eng..

[24] Wagner S.C., Hewison T., Stone T., Lachérade S., Fougnie B., Xiong X. (2015). A summary of the joint GSICS – CEOS/IVOS lunar calibration workshop: Moving towards intercalibration using the Moon as a transfer target. Proceedings SPIE 9639, Sensors, Systems, and Next-Generation Satellites.

[25] Thuillier G., Hersé M., Labs D., Foujols T., Peetermans W., Gillotay D., Simon P., Mandel H. (2003). The Solar Spectral Irradiance from 200 to 2400 nm as Measured by the SOLSPEC Spectrometer from the Atlas and Eureca Missions. Sol. Phys..

[26] D X., Xu N., Hu X.Q., Wu R.H., Niu X.H., Wang X.H., He Y.Q. (2020). On-Orbit Detection and Correction of Crosstalk Effect of FY-3D MERSI-II Signals. Acta Opt. Sin..

[27] Xu N., Wu R.H., Hu X.Q., Chen L., Wang L., Sun L. (2015). Integrated Method for On-Obit Wide Dynamic Vicarious Calibration of FY-3C MERSI Reflective Solar Bands. Acta Opt. Sin..

[28] Ma L., Zhao Y., Woolliams E., Dai C., Wang N., Liu Y., Li L., Wang X., Gao C., Li C. (2020). Uncertainty Analysis for RadCalNet Instrumented Test Sites Using the Baotou Sites BTCN and BSCN as Examples. Remote Sens..

